# Advanced Antigen Delivery of Murine Survivin: Chimeric Virus-Like Particles in Cancer Vaccine Research

**Published:** 2007-09

**Authors:** Thomas Schumacher, Claus Ruehland, Christine Schultheiss, Marc Brinkman, Franz Roedel, Christian O. A. Reiser, Juergen Hess, Christoph Reichel

**Affiliations:** 1 *Siegfried Biologics GmbH, Heinrich-Hertz-Str. 1b, 14532 Kleinmachnow, Germany;*; 2 *Department of Radiation Therapy, Universitaetsstr. 27, 91054 Erlangen, Germany;*; 3 *TRION Pharma, Frankfurter Ring 193a, 80807 Munich, Germany;*; 4 *Com4Doc, Am Klopferspitz 12, 82152 Martinsried, Germany*

**Keywords:** cancer, vaccines, virus-like particles, cell-mediated immunity, survivin

## Abstract

Success in cancer immunotherapy depends on the identification and efficient targeting of specific tumor-associated antigens. Two pivotal strategies to prime patients’ immune system against malignant cells are tumor-specific adoptive T-cell therapy and tumor-specific vaccination. Here, we will focus on immunotherapeutic vaccination and discuss the advantages and disadvantages of different strategies to deliver tumor-specific T-cell epitopes. A particular focus will be put on virus-like particles (VLPs) as vehicle to deliver tumor-specific epitopes in the context of full-length proteins, as multi-epitope constructs or as individual tumor-associated T-cell epitopes. VLPs represent non-infectious and non-replicating antigen delivery systems devoid of any nucleic acid. They constitute innovative immunotherapeutic agents against cancer due to their superior, adjuvant-like antigenicity. We will present various tumor-associated antigens currently in different stages of development including survivin, as promising candidates for targeted tumor therapies.

## INTRODUCTION

**Figure 1:**
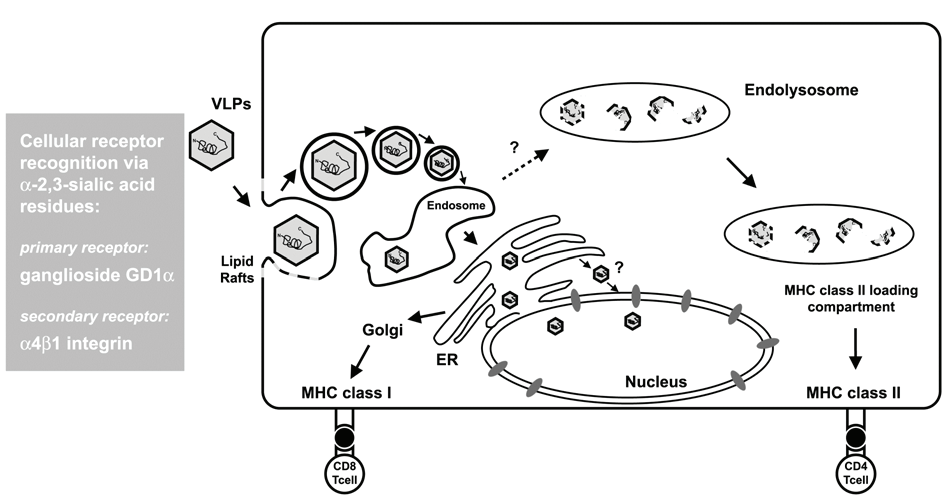
Model of the cellular entry of VLPs and subsequent antigen presentation. Within antigen presenting cells (APCs) the endosome-mediated internalization pathway for antigens delivered by polyomavirus VLPs, coincides with the TAP-dependent MHC class I-specific antigen processing and presentation process of the ER. Therefore, MHC class I-restricted CD8 T cells are preferentially induced by VLP-based immunization. Alternatively, VLP vaccination may also lead to the stimulation of MHC class II-restricted CD4 T cells via antigen uptake along the endosomal-lysosomal pathway to MHC class II-loading compartments of APCs. Cellular receptors are recognized via α-2,3-sialic acid residues, with ganglioside GD1 and 1-integrin being the primary and secondary receptors.

Successful treatment of cancer is believed to strongly rely on antigen-specific CD8^+^ cytotoxic T lymphocyte (CTL) responses. Antigen-derived peptides associated with major histocompatibility complex (MHC) class I molecules are presented on the surface of antigen presenting cells (APCs) such as macrophages and dendritic cells (DCs). To stimulate CD8 T, cells soluble exogenous antigens can access the cytosol of these APCs through the endosome-mediated internalization pathway or alternatively through the TAP-dependent MHC class I-specific antigen processing and presentation pathway (Fig. 1).

For the generation of more efficacious CTL responses elicited by immunotherapeutic cancer vaccines, the appropriate antigen availability for MHC class I presentation has to be improved further. Several strategies were developed for enhancing antigen delivery into the cytosol of APCs. These vaccination strategies include viral vectors like the ones based on alphaviruses or lentiviruses with strong immunodominance capable of introducing antigens into MHC class I processing and presentation pathways (1-4). Other approaches rely on packaging of tumor-associated antigens as cargo into lipid-based vesicles such as liposomes or virosomes (5, 6). However, these are hampered by the rather poor immunogenicity of the lipid particles themselves, or based on antigen delivery through bacterial secretion systems (7, 8). Protein-based virus-like particles (VLP) receive growing attention due to their capacity to elicit cell-mediated immunity (CMI) with emphasis on CD8 T cell responses and due to their lack of infectivity.

**Figure 2:**
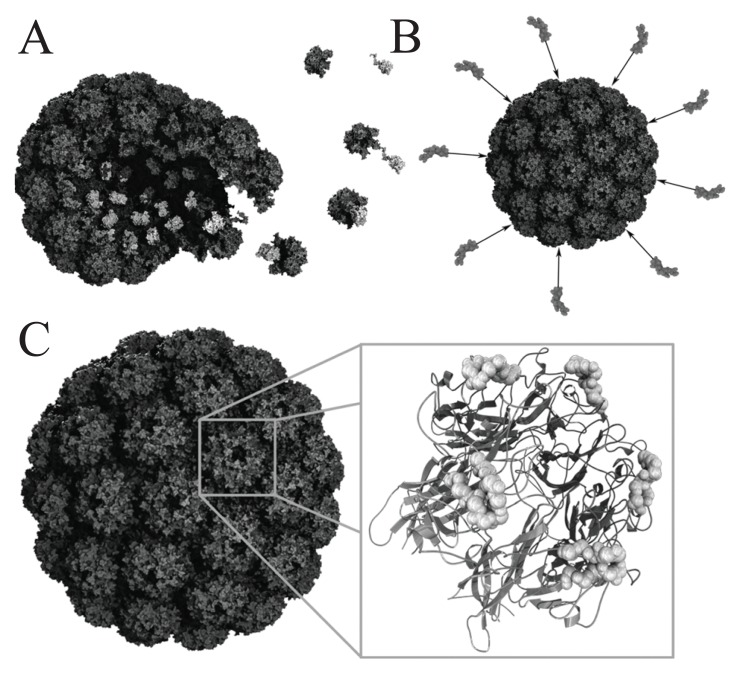
Schematic view of polyomavirus VLP assembly and modes of antigen presentation. (A) Assembly of polyomavirus VLPs from 72 VP1 pentamers (dark gray) and VP2 (light gray). VP2 monomers associate with pentamers via hydrophobic interactions between the inner surface of the pentamer structures and the VP2 C-terminus. (B) Antigen presentation may be achieved via chemical cross-linking of B- or T- cell epitopes onto the surface of pre-assembled particles. (C) Detailed view of polyomavirus VLP (left) and a 3-D model of a single VP1 pentamer depicted as ribbon model calculated from 3-D structural data (NCBI: 1CN3) (right). Surface-exposed BC2 loop superimposed, with peptide backbone presented as ball model. This loop may be used for the insertion of B- or T- cell epitopes.

VLPs are nanosized particulate structures consisting of protein-based subunits derived from viruses (Fig. 2) (4, 9). At present, vaccine development utilizing VLPs from the hepatitis B surface antigen or human papillomavirus (HPV) L1 is mainly focused on the induction of neutralizing antibodies (10). An HPV VLP vaccine was recently approved (Gardasil, Merck & Co, Inc.) and a hepatitis B VLP vaccine is already on the market for more than a decade. Thus, the success story of this unusual class of protein-based vaccines is just getting started. Currently, several research advances unveil new applications of chimeric VLPs in cancer vaccine development, which requires cell-mediated immune responses (Table 1). VLPs possess *per se* adjuvant activity stimulating innate and cell-mediated immunity not only to the carrier (VLP), but also to the tumor-associated self antigens displayed by the particles. Based on the inherent capacity to break self-tolerance, VLPs represent ideal antigen delivery devices for immune intervention in preventive and therapeutic settings (4, 9-11).

**Table 1:** Selected cVLPs stimulating cell-mediated immunity in the field of vaccine research

Foreign antigens	VLP carriers	Comments	References

E7 full-length (HPV16)	L1/L2 (HPV)	Induction of protective CD8 T cell responses against TC-1 tumour challenge	Greenstone *et al*. (12)
LCMV Epitope 118-132	VP2 VLP (parvovirus)	Induction of protective CD8 T cell responses against LCMV challenge	Sedlik *et al*. (34)
Ovalbumin Epitope 257-264	VP1/VP2 (polyoma)	Induction of beneficial CD8 T cell responses in preventive or therapeutic settings against murine melanoma (B16 and B16-OVA)	Brinkman *et al*. (19)
TRP2 Epitope 180-192			Brinkman *et al*. (20)
GFP full-length	VP1/VP2 (polyoma)	Induction of protective and therapeutic immune responses against B16-GFP melanoma	Abbing *et al*. (18), Boura *et al*. (22), Reichel *et al*. (9)
HER2 (1-683)	VP1/VP2 (polyoma)	Induction of protective T cell responses against D2F2/E2 mammary carcinoma; beneficial treatment of BALB-neuT mice	Tegerstedt *et al*. (21)
LCMV p33 peptide	HBcAG (HBV)	Eradication of established solid fibrosarcoma tumours; protection against LCMV challenge and recombinant vaccinia infection	Storni *et al*. (35), Storni *et al*. (36)

## INDUCTION OF CELL-MEDIATED IMMUNITY BY VIRUS-LIKE PARTICLES

In an early example human papillomavirus (HPV)-based VLPs induced protective CD8 T cell responses in an HPV16 tumor model (12). HPV VLPs consisting of the viral structural subunits L1 and L2, with L2 fused to the viral tumor antigen E7 (L2-E7), are capable of stimulating prominent CMI in relevant animal models (Table 1). Greenstone and colleagues (12) induced VLP-mediated protection against tumor challenge in MHC class II-deficient C57BL/6 mice by using the tumor cell line TC-1, which expressed the immunodominant HPV16 E7 antigen. While these chimeric VLPs triggered protection against tumor challenge in this one mice strain, they did not in beta2-microglobulin or perforin knockout mice, implying that protection was mediated by MHC class I-restricted cytotoxic T cells (12).

In a similar approach dihydrofolate reductase (DHFR) from *E. coli* was inserted into a surface exposed loop of murine polyomavirus VP1 (Fig. 2). The formation of pentamers and the assembly into capsoids was unaffected, although DHFR showed reduced thermal stability, but proved to be enzymatically active with only slightly reduced substrate affinities (13). Further, full-length insertion of the green fluorescent protein (GFP) was demonstrated for the hepatitis B virus-nucleocapsoid HBcAg, which contains potent T helper epitopes. The immunodominant c/e1 epitope located at the tips of prominent surface spikes of the particle, was used as insertion site and fluorescent particles were efficiently formed (14). The induction of GFP-specific antibodies via HBcAg/GFP immunization indicates that GFP antigens were surface-exposed. While these examples of presentation of full-length, active proteins are promising, the antigen delivery by means of introducing complete proteins into surface-exposed domains has to be further evaluated by the insertion of more flexible foreign proteins to see whether these may hamper or even inhibit particle formation. Since GFP represents a very compactly folded, barrel-shaped molecule, the current findings have to be considered with care. It is rather likely that approaches based on the insertion of proteins into surface-exposed loops will in several - if not many - cases affect assembly of VLPs, and therefore this concept may not be generally suited as robust antigen delivery approach.

To bypass these structural limitations of the delivery of antigens in the form of complete proteins by VLPs, murine polyoma VLPs harboring a protein-binding domain (WW domain) of the mouse formin binding protein 11 (FBP11) were employed. These WW domains interact with proline-rich ligands containing a PPLP motif (15). PPLP-tagged peptides or proteins (like GFP) are packaged inside these modified VLPs, since the N-termini of VP1 subunits are not surface-exposed, but rather lie within the interior of these particles (15, 16). This approach limits the structural constrains for the assembly process of the modified VP1 monomers carrying the WW domain. Any cargo packaged into these VLPs by interaction with the WW domain would likely have only limited or no additional negative effect on the assembly of the particles.

Another way to hide foreign proteins within the cavity of polyomavirus VLPs utilizes the naturally occurring hydrophobic interaction between the capsid proteins VP1 and VP2/VP3 (Fig. 2) (17). Several reports demonstrate the successful expression and assembly of chimeric VLPs with different proteins and epitopes internalized into these particles as fusion proteins to VP2/VP3 derivatives (4, 9, 18-22).

Whether structurally complex interplay between heterologous ligands and binding domains or the native self-assembly of VP1 and VP2/VP3 capsid proteins – it needs further clarification what is best suited for the interior packaging of protein antigens into, or surface loading onto polyoma VLPs. And in the end, it still may be determined by the character of the tumor-associated antigen. In regard of the industrial manufacturing of VLPs in yeast, the latter notion may represent the most plausible strategy for accomplishing high yields of heterologous particles directly isolated from *S. cerevisiae*, due to the simplicity of the manufacturing process.

The natural polyomavirus co-assembly process has already been utilized in several studies. Using an *Escherichia coli*-based expression system Abbing and colleagues (18) pinpointed the VP2 domain interacting with VP1 to a stretch of 43 amino acids (VP2 _anchor_). A GFP-VP2_anchor_ fusion protein was co-assembled *in vitro* into VP1 VLPs reaching 89% of the theoretical packaging efficiency, assuming 72 VP1 pentamers per nanoparticle. After subcutaneous (s.c.) tumor implant with 10^5^ B16-GFP melanoma cells, mice were s.c. treated with 50 μg VLPs VP1/GFP-VP2_anchor_ at day 4 and 11 leading to a 60% survival rate (9). It should be noted that in the preventive setting GFP-containing VLPs induced protection rates of 80% against B16-GFP melanoma challenge (9). Without vaccination or treatment, more than 50% of mice died within 20 days after tumor cell implantation. This demonstrated that the natural hydrophobic interaction between VP1 and VP2/VP3 is perfectly suited for the packaging of cargo proteins into polyomavirus VLPs, and that these VLPs retain the capacity to induce strong anti-tumoricidal responses.

To assess the capacity of murine polyoma VLPs to prime CD8 T cell responses, a protein fragment harboring the immunodominant CD8^+^ cytotoxic T cell epitope of ovalbumin (OVA_257-264_), was fused to the C-terminus of VP1 resulting in a VP1-OVA_252-270_ fusion protein (20). Heterologous VP1-OVA_252-270_ pentamers expressed in *E. coli*, were assembled into VLPs *in vitro* under high salt conditions. These VLPs were analyzed for their capacity to elicit OVA_257-264_ CD8 T cell responses *in vivo* and to protect mice against lethal thymoma or melanoma challenge. Two s.c. applications of heterologous VP1-OVA_252-270_ VLPs given in weekly intervals induced protective immunity against OVA-expressing tumor cell lines (19, 20). Beneficial therapeutic effects were also reported for particulate VP1 structures harboring a CTL epitope taken from the self antigen tyrosinase-related protein 2 (TRP2) in a melanoma model (19). The use of peptides derived from TRP2 that is differentially expressed in melanoma cells and melanocytes provides a rodent model that closely mimics human melanoma without introduction of xenogenic or otherwise foreign antigen (19), clearly demonstrating the ability of this particles to break self-tolerance.

Likewise, this delivery concept was used to package the tumor antigen Her2/neu into murine polyomavirus VLPs, again exploiting the natural hydrophobic interaction (Fig. 2) between VP1-pentamers and VP2 (21). VLPs were assembled from VP1 and a fusion protein between full-length VP2 and the extracellular and transmembrane domain of HER2/neu_1-683_. These VLPs were employed as vaccine against HER-2/neu-expressing tumors in animal models. A single subcutaneous application of 50 μg HER2/neu_1-683_ VLPs evoked a complete rejection of HER2/neu-positive D2F2/E2 mammary carcinoma cells in BALB/c mice. Data from ELISPOT assays indicate that the observed protective immunity was most likely due to cell-mediated immune responses. Antibodies against HER2/neu were not detected. It was estimated that only one single molecule of the VP2-HER2/neu_1-683_ fusion protein (~110 kDa in size) was packaged into each 45 nm VP1-particle. These VP2-HER2/neu_1-683_-containing VLPs were manufactured in insect cells using a baculovirus expression system.

## VLPS DELIVERING MURINE SURVIVIN AS TUMOR-ASSOCIATED ANTIGEN

Survivin belongs to a family of proteins, known as inhibitor of apoptosis protein (IAP), which plays a key role in the regulation of apoptosis and cell division (24). As compared to normal differentiated tissue, survivin is abundantly expressed in the embryonic development and in most tumors, making it an ideal universal target antigen for immunotherapeutic intervention against cancer. Recently, the therapeutic effectiveness of CTL epitopes derived from survivin was demonstrated both in mice and human (25-29).

**Figure 3:**
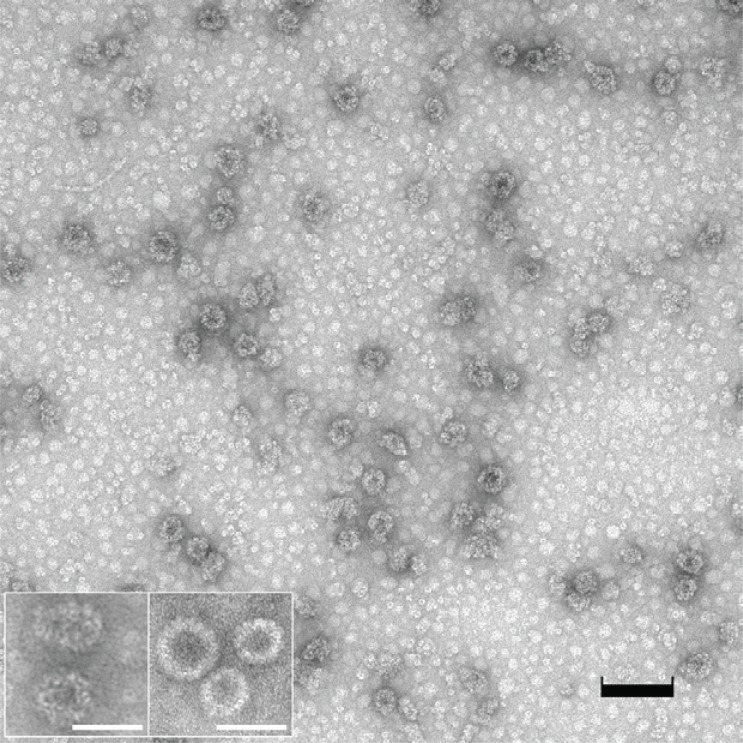
Electron microscopic view of VP1-survivin VLPs assembled *in vitro*. The VLP preparation was transferred to carbon-coated Formar copper grids (300 mesh) and stained with 5% uranyl-acetate. The preparation contained multiple irregularly shaped VLPs as well as pentameric structures (black bar=100 nm). The inset depicts (left) VP1-survivin VLPs at higher magnification, and (right) regularly shaped VP1 VLPs expressed in and purified from the yeast S. cerevisiae (bars=50 nm).

In order to evaluate survivin as tumor antigen delivered by VLPs, full-length murine survivin was expressed in *E. coli* as C-terminal translational fusion of the polyoma VP1 capsid protein. Pentameric structures that are known to be formed in *E. coli* were assembled into VLPs (Fig. 3). VLP preparations contained a considerable proportion of non-assembled VP1-survivin pentamers and a rather heterogeneous population of VLPs and VLP-like structures. Correctly sized VLPs (approx. 45 nm diameter) were characterized by irregularly shaped outer rims (Fig. 3), which indicate that the large C-terminal fusion (16.4 kDa) interfered with correct assembly. Nevertheless, these preparations were subsequently used for preventive and therapeutic immunizations, since it was previously shown that even pentamers exhibit a strong immunogenic capacity, only slightly lower than that of correctly assembled VLPs (4, 9, 30, 31). The fact that the C-terminal fusion of murine survivin to VP1 affected correct assembly has direct implications for the strategy of delivery of tumor-associated antigens via VLPs. While in the case of survivin at least partial assembly was achieved, it is conceivable that each tumor-associated antigen will affect assembly in its own way, i.e. there will be many tumor-specific antigens that will exclude themselves from this strategy of antigen presentation and delivery simply because assembly will be hampered. Therefore, approaches employing the natural hydrophobic interaction between VP1 pentamers and VP2/VP3-fused tumor-associated antigens may be advantageous and will probably lead to less heterogeneous VLP preparations (see previous sections).

## PREVENTIVE AND THERAPEUTIC IMMUNIZATION WITH HETEROLOGOUS VLPS CARRYING MURINE SURVIVIN AGAINST MELANOMA

**Figure 4:**
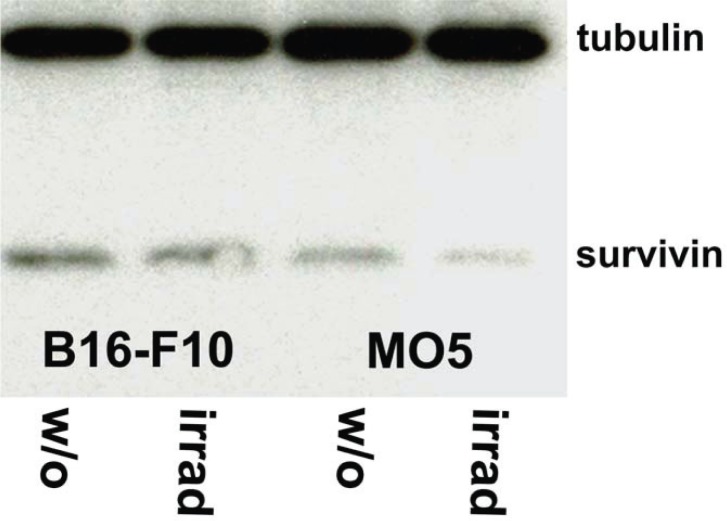
Expression levels of survivin are independent of irradiation-induced apoptosis. The two survivin expressing cell lines B16-F10 and MO5 were irradiated for the induction of apoptosis. Aliquots of non-irradiated and irradiated cells were washed and lysed in RIPA buffer supplemented with a protease inhibitor cocktail. Equal amounts of protein (10 μg) were subjected to SDS-PAGE and Western blot analysis with anti-survivin and anti-tubulin antibodies. All four samples contained approximately equal amounts of protein as confirmed by the anti-tubulin labelling, and expressed comparable amounts of survivin, indicating that the survivin expression levels were independent of induction of apoptosis.

Prior to analyzing the therapeutic potency of VP1-survivin VLPs, naive expression of survivin and expression of survivin after induction of apoptosis in two melanoma cell lines, was investigated. To induce apoptosis B16F10 and MO5 cells are irradiated. Preparations of the cells were analyzed with anti-survivin and anti-tubulin antibodies (Fig. 4).

**Figure 5:**
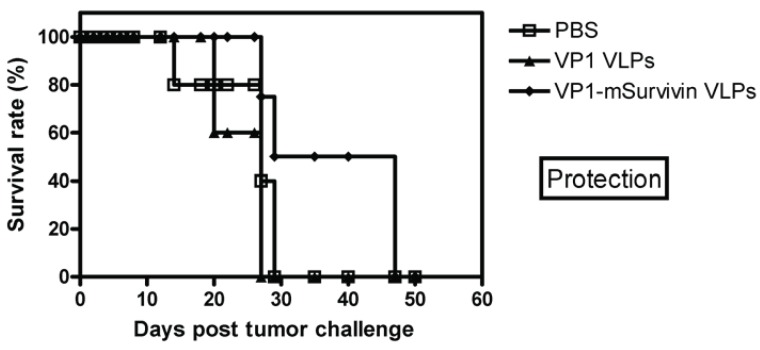
Protection with VP1-mSurvivin VLPs in a mouse melanoma model. Four C57BL/6 mice per group were immunized twice with VP1-mSurvivin VLPs in 7 days intervals and were charged 7 days later s.c. with 105 MO5 melanoma tumor cells. One group was mock-immunized with PBS (open squares), the next with VLPs lacking the tumor antigen (solid triangles) and the last group with VP1-mSurvivin VLPs (solid diamonds). Immunization with antigen-presenting VLPs significantly increased the survival rate of the animals.

Subsequently, C57BL/6 mice were subcutaneously immunized with VP1-survivin VLPs. Controls were mock-immunized with buffer or vaccinated with VLPs lacking the tumor-associated antigen. Seven days later these mice were inoculated with MO5 melanoma cells. The survival rate of the animals was monitored over a period of 50 days. Mice that had received VP1-survivin VLPs showed significantly increased survival within the observation period (Fig. 5). This demonstrates the therapeutic value of VP1-survivin VLPs despite the hampered assembly capacity and irregular shape of the particles. Further, this confirms the ability of these particles to break self-tolerance of the endogenously expressed tumor-associated antigen survivin.

**Figure 6:**
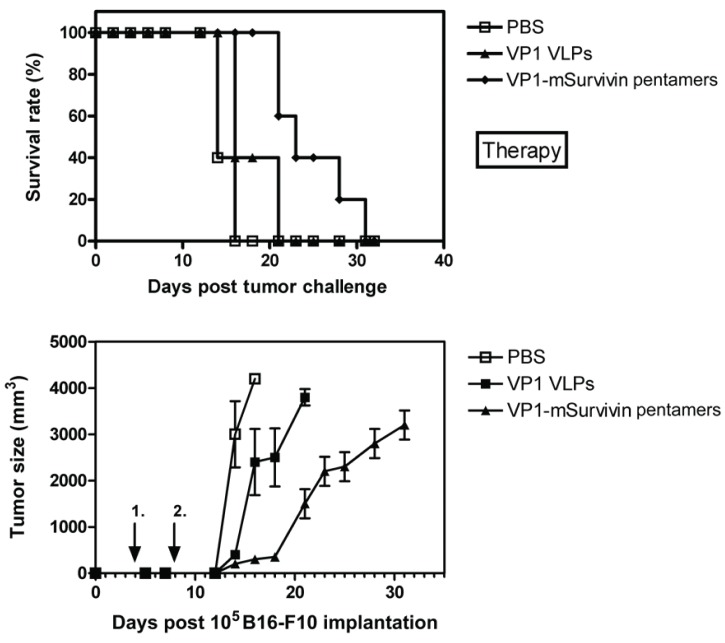
Therapy and tumor growth with VP1-mSurvivin pentamers in a mouse melanoma model. Five C57BL/6 mice per group were charged s.c. with 10^5^ B16-F10 melanoma tumor cells and subsequently immunized twice with VP1-mSurvivin pentamers after 4 and 8 days. One group was mock-immunized with PBS (open squares), the next with pentamers lacking the tumor antigen (solid triangles) and the last group with VP1-mSurvivin pentamers (solid diamonds). (A) Immunization with antigen-presenting pentamers significantly increased the survival rate of the animals. (B) As expected, tumor growth was clearly delayed by the immunization with antigen-presenting pentamers.

In a similar experiment C57BL/6 mice were charged s.c. with B16-F10 melanoma tumor cells and subsequently immunized with VP1-survivin pentamers. Immunization with antigen-presenting pentamers significantly increased the survival rate of the animals in this therapy approach (Fig. 6a). As expected, tumor growth was clearly delayed by the immunization with antigen-presenting pentamers (Fig. 6b).

This demonstrates that particulate VLP structures displaying the full-length murine survivin antigen as C-terminal VP1 fusion have beneficial immunotherapeutic effects under early onset of treatment measures in a murine melanoma model.

## CONCLUDING REMARKS

The most important concerns with novel CTL-stimulating vaccines capable of introducing antigens into MHC class I processing and presentation pathways is represented by safety issues seen with replicating vectors. Therefore, the development of non-replicating antigen delivery systems is an important advance in the design of efficacious and safe CTL-priming cancer vaccines. In this regard VLPs receive growing attention due to their capability to elicit CMI with emphasis on CD8 T cell responses in the absence of additional immunostimulatory substances.

The fact that chimeric VLPs, which represent important antigen sources for MHC class I-specific processing and presentation pathways, are efficiently taken up by DCs *in vivo* (22, 32, 33) has important implications for the development of vaccines based on such VLPs carrying complete protective self or non-self antigens, fragments of these polypeptides or CTL epitopes of the respective proteins. Since VLPs and even pentameric structures are able to break T cell tolerance and since both antigen-specific CD4 and CD8 T cells are required for CMI, heterologous VLP-based vaccines delivering complete antigens or appropriate protein fragments will offer new therapeutic opportunities in the treatment of cancer or chronic infectious disease. By using universal tumor antigens expressed by specific types of tumor cells originating from a multitude of cancer patients, this novel vaccine generation may significantly advance this field. The major advantage of these outstanding antigen delivery systems administered in the absence of adjuvants is that protein-based nanoparticles should be well tolerated and safe even in immunocompromized individuals.
